# Supplementary material for: Volumetric grey matter alterations in adolescents and adults born very preterm suggest accelerated brain maturation
Source: Neuroimage. 2017 Dec;163:379–89. doi: 10.1016/j.neuroimage.2017.09.039 (PMC5725310; doi:10.1016/j.neuroimage.2017.09.039)
Supplement: Supplementary file 1 [file mmc1.docx]

**Karolis, V. R., Froudist-Walsh, S., Kroll, J., Brittain, P. J., Tseng, C.-E., Nam, K.-W., Reinders, A. A. T. S., Murray, R. M., Williams, S. C. R., Thompson, P.M., & Nosarti, C.**

**Volumetric grey matter alterations in adults born very preterm suggest accelerated brain maturation**

**Supplementary Materials.**

Table S1. Characteristics of the normative data set


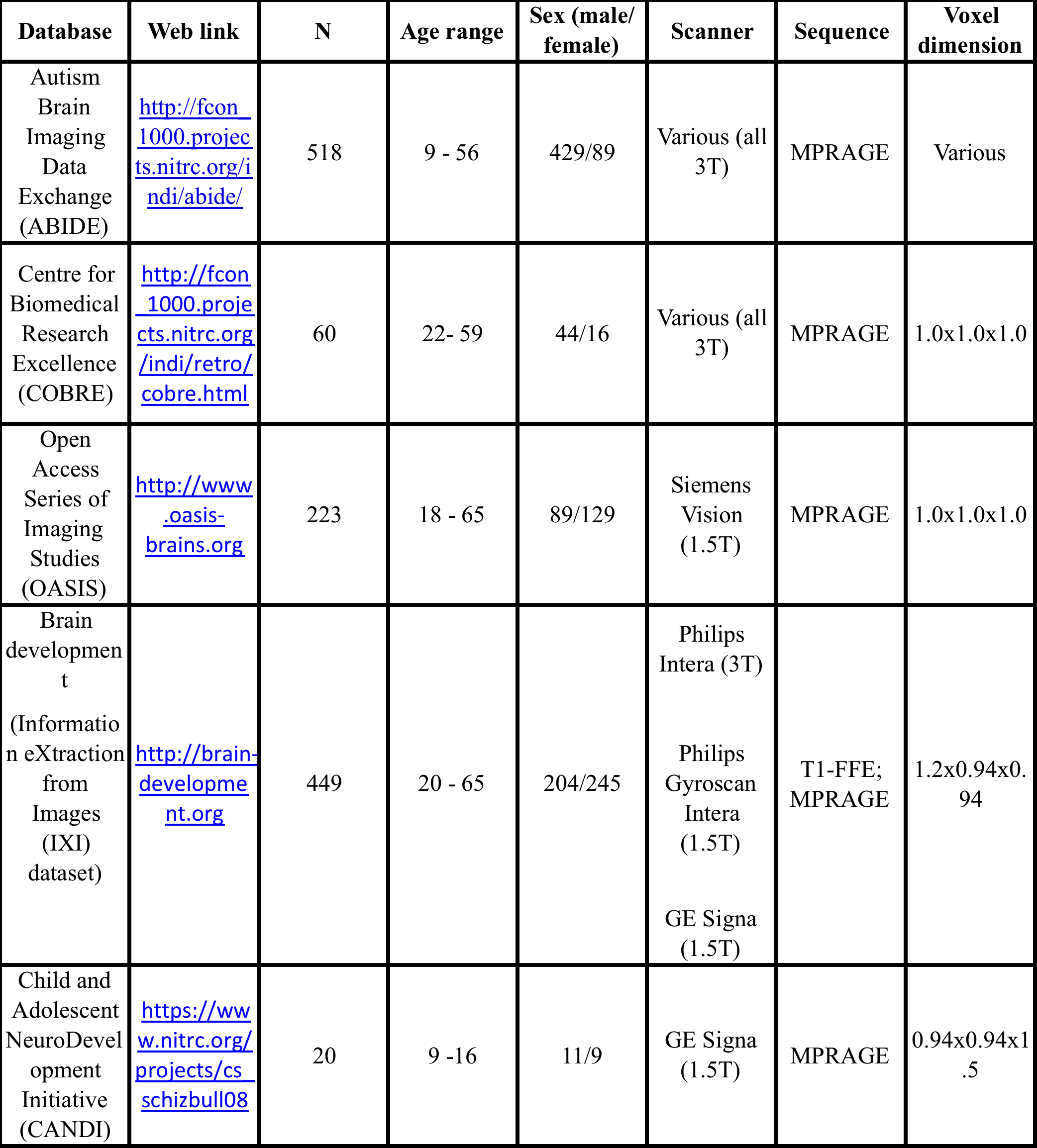


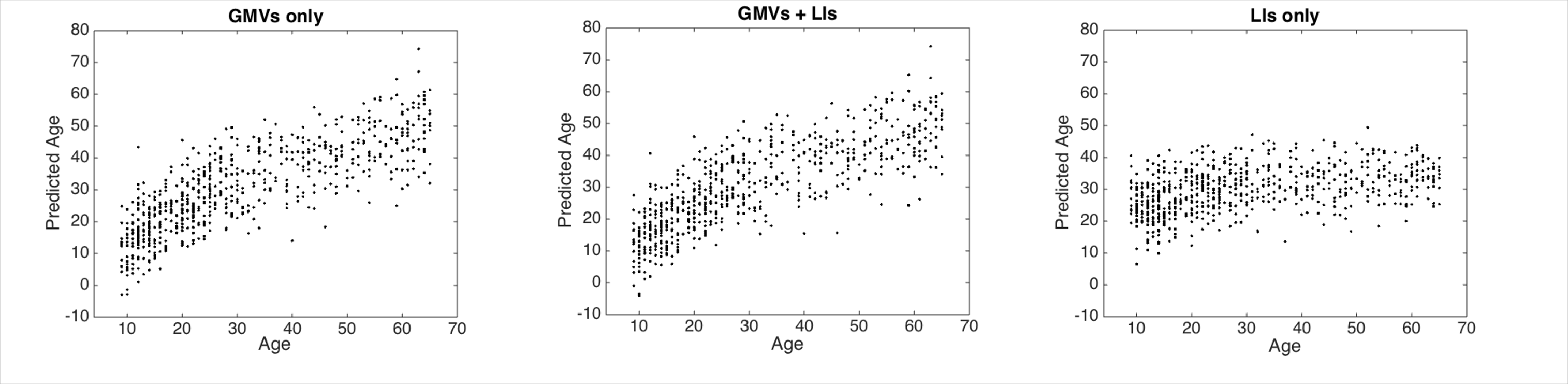


Figure S1. Age prediction in the normative data set (split-half) using: GMVs markers only (left panel), GMVs +LIs markers (middle panel) and LIs only (right panel).

**Creating hierarchy of structural markers**

Feature re-parameterisation was performed as follows:

1. Two types of volumetric markers were obtained: (a) the mean of homologous regions in the left and right hemisphere, hereafter - regional GMVs; (b) the difference between homologous regions in the left and right hemisphere - hereafter - regional lateralisation indices (LIs). The further steps refer to GMVs, but were exactly the same for LIs.
2. GMVs were combined into two *N* x 41 feature matrices, one for the normative data set and one for the study sample, where *N* is the number of participants in a sample (either 1270 or 560) and 41 is the number of GMVs.
3. A 41x41 correlation matrix was calculated for the normative dataset, so that each element of the matrix reflected the correlation between GMVs in a pair of distinct brain regions across subjects.
4. Modularity analysis was run on the above correlation matrix, to subdivide the entire set of brain regions into modules. Modular partitioning was obtained using the Louvain method for community detection (Blondel et al. 2008). As certain heuristics are embedded in the algorithm, the partitioning was performed 1000 times (Dwyer et al. 2014). A matrix was generated on each algorithm iteration, where element (*i, j*) assumed the value of 1 if the nodes *i* and *j* were designated to the same module; otherwise it assumed the value of 0. Resulting matrices were averaged across iterations to obtain a consistency matrix, which was submitted again to the Louvain process for a final partitioning into modules.
5. Following partitioning, the original feature matrices of each data set were divided into submatrices, **R_1_**, …, **R_k_**, such that any **R_i_** combined the regional GMVs that belonged to a particular module, and *k* was the number of modules identified by modularity analysis.
6. A principal components analysis (PCA) was performed on the normative data set. The first principal component, **PC-a^i^**, was calculated for each module independently, i.e., for each **R_i_** in the normative dataset. We call this stage PCA-A, to distinguish it from a later stage in the analysis.
7. Individual data points (i.e., the rows of **R_i_**) in both data sets were projected onto **PC-a^i^**. Subject loadings along **PC-a^i^** were taken to represent GVM for module *i*.
8. Each **R_i_** in both data sets was then residualised with respect to **PC-a^i^**. Each column **r_i_** of a residualised **R_i_** was taken to represent a region-specific GMV (see **Figure 1*A***).
9. – 12) Steps (5)–(9) were repeated in the next hierarchical level of analysis, PCA-B, but this time for modular GMVs (i.e., subject loadings along all **PC-a^i^**’s) which were considered as members of one, global, module. In this way, we obtained global and module-specific GMVs, **g**, and **m_i_**, respectively (see **Figure 1*A-B***).

**Procedure for choosing random effects in the mixed-effect models**

Three random effects, modelling the within-participant covariance structure, were evaluated: 1) intercept only, 2) intercept and participants’ age (centred on the grand mean) combined and 3) intercept, participants’ age and participants’ age squared combined. A log-likelihood model comparison test demonstrated that the set of random factors that included intercept and participants’ age outperformed the intercept model in all but one case. It also outperformed the model that included intercept, participants’ age and participants’ age squared in 80% of cases. Consequently, the intercept and linear effect of age were retained as random factors in all subsequent statistical analyses.

**Handedness and LI**

Even though handedness did not statistically differ between the groups, we explicitly accounted for its effect in LI analysis because of a higher prevalence of missing data in the control compared to the very preterm group. All data were included in this analysis, with missing values treated as a separate level of handedness. Results showed two discrepancies compared to the previous findings. Firstly, the relative greater right lateralisation in putamen in the very preterm group was no longer significant. Instead, Module #5, which comprised basal ganglia structures (including putamen), anterior cingulate gyrus, Heschl’s gyrus and insula, showed a relative greater right lateralisation in the very preterm group compared to controls (β = -.33, CI =[-.58 -.08]).

**Specificity of association between Maturation Index (MI) and gestation age (GA)**

Given that the predicted participants’ age represents a linear combination of GMVs, weighted by coefficients of the age-predictive model, we also investigated the specificity of its association with GA, i.e. we wanted to investigate whether this association could be accounted for by collinearity between MI and some arbitrary linear combination of GMVs. To do this, we generated 20000 linear combinations of GMVs by applying random weighting in the range of -1 to 1. We then selected 1000 (5%) linear combinations, which showed the highest absolute correlation with MI. We then residualised MI with respect to each of them and fitted the models to each residualised MI in order to obtain a mean and standard deviation of regression coefficient associated with GA.

**Figure S2** shows the mean and standard deviation of standardised regression coefficients associated with GA obtained in the analysis. We also plotted the coefficients obtained after residualising with respect to a set of preselected (‘meaningful’) GMVs linear combinations. In all cases, the means of these coefficients were within 1 standard error of the coefficient fitted to the unresidualised MI.


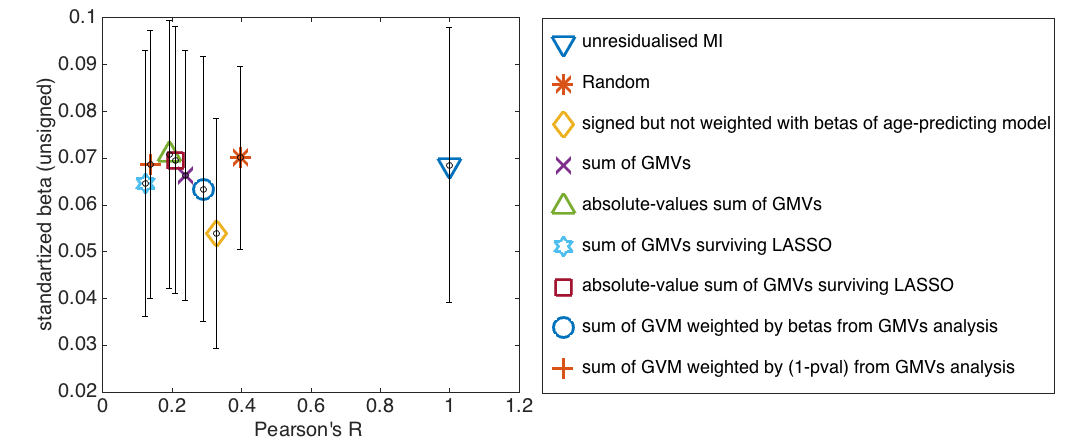


Figure S2. Association between GA and MI following residualisation of MI with respect to a set of GVM linear combinations, as detailed in figure legends. On x-axis, a correlation between MI and a linear combination used to residualise MI.
